# (Thio­cyanato-κ*S*)­tris­(thio­urea-κ*S*)mercury(II) chloride

**DOI:** 10.1107/S1600536813011847

**Published:** 2013-05-04

**Authors:** A. Shihabuddeen Syed, K. Rajarajan, M. NizamMohideen

**Affiliations:** a Research and Development Center, Bharathiar University, Coimbatore 641 046, India; b Department of Physics, Rajeswari Vedachalam Government Arts College, Chengalpet 603 001, India; c Department of Physics, The New College (Autonomous), Chennai 600 014, India

## Abstract

In the title salt, [Hg(NCS)(CH_4_N_2_S)_3_]Cl, the Hg^2+^ ion is coordinated in a severely distorted tetra­hedral manner by three thio­urea groups and one thio­cyanate anion through their S atoms. The S—Hg—S angles vary widely from 87.39 (5) to 128.02 (4)°. Weak intra­molecular N—H⋯S hydrogen bonds are observed, which form *S*(6) ring motifs. In the crystal, the ions are linked by N—H⋯N and weak N—H⋯Cl inter­actions, generating a three-dimensional network.

## Related literature

For background to mercury(II) complexes with thio­urea and thio­cyanate ligands, see: Nawaz *et al.* (2010 ▶). For hard and soft acids and bases, see: Ozutsmi *et al.* (1989 ▶); Bell *et al.* (2001 ▶). For related structures, see: Safari *et al.* (2009 ▶); Nawaz *et al.* (2010 ▶); Ramesh *et al.* (2012 ▶). For graph-set notation, see: Bernstein *et al.* (1995 ▶).

## Experimental

### 

#### Crystal data

[Hg(NCS)(CH_4_N_2_S)_3_]Cl
*M* *_r_* = 522.49
Orthorhombic, 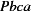

*a* = 8.2175 (3) Å
*b* = 16.3257 (8) Å
*c* = 22.6793 (10) Å
*V* = 3042.6 (2) Å^3^
*Z* = 8
Mo *K*α radiation
μ = 10.83 mm^−1^
*T* = 293 K
0.30 × 0.25 × 0.20 mm

#### Data collection

Bruker Kappa APEXII CCD diffractometer
Absorption correction: multi-scan (*SADABS*; Sheldrick, 2004 ▶) *T*_min_ = 0.140, *T*_max_ = 0.221
38987 measured reflections
5125 independent reflections
3579 reflections with *I* > 2σ(*I*)
*R*_int_ = 0.057

#### Refinement

*R*[*F* ^2^ > 2σ(*F* ^2^)] = 0.036
*wR*(*F* ^2^) = 0.082
*S* = 1.05
5125 reflections
155 parameters
H-atom parameters constrained
Δρ_max_ = 2.17 e Å^−3^
Δρ_min_ = −1.21 e Å^−3^

### 

Data collection: *APEX2* (Bruker, 2004 ▶); cell refinement: *APEX2* and *SAINT* (Bruker, 2004 ▶); data reduction: *SAINT* and *XPREP* (Bruker, 2004 ▶); program(s) used to solve structure: *SHELXS97* (Sheldrick, 2008 ▶); program(s) used to refine structure: *SHELXL97* (Sheldrick, 2008 ▶); molecular graphics: *ORTEP-3* for Windows (Farrugia, 2012 ▶) and *Mercury* (Macrae *et al.*, 2008 ▶); software used to prepare material for publication: *WinGX* (Farrugia, 2012 ▶) and *PLATON* (Spek, 2009 ▶).

## Supplementary Material

Crystal structure: contains datablock(s) global, I. DOI: 10.1107/S1600536813011847/jj2165sup1.cif

Structure factors: contains datablock(s) I. DOI: 10.1107/S1600536813011847/jj2165Isup2.hkl

Additional supplementary materials:  crystallographic information; 3D view; checkCIF report

## Figures and Tables

**Table 1:** Hydrogen-bond geometry (Å, °)

*D*—H⋯*A*	*D*—H	H⋯*A*	*D*⋯*A*	*D*—H⋯*A*
N1—H1*A*⋯Cl1^i^	0.86	2.48	3.277 (4)	155
N1—H1*B*⋯S2	0.86	2.76	3.475 (5)	142
N2—H2*A*⋯Cl1^i^	0.86	2.55	3.335 (4)	152
N3—H3*B*⋯Cl1	0.86	2.61	3.320 (5)	141
N4—H4*B*⋯Cl1^ii^	0.86	2.51	3.370 (5)	175
N5—H5*B*⋯Cl1	0.86	2.54	3.363 (4)	161
N5—H5*A*⋯N7^iii^	0.86	2.11	2.933 (7)	160

Symmetry codes: (i) 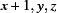; (ii) 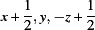; (iii) 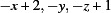.

**Fig. 1.**
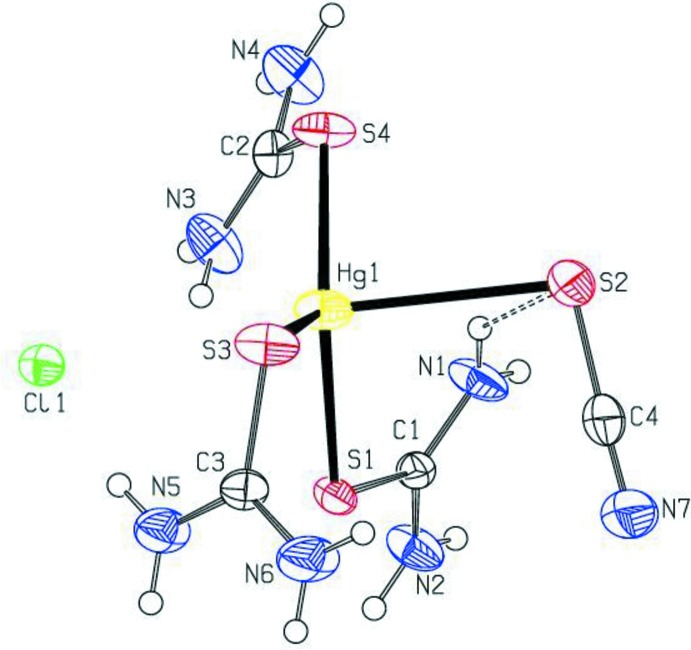
View of the title compound showing the atom-numbering scheme. Displacement ellipsoids are drawn at the 40% probability level. H atoms are presented as a small spheres of arbitrary radius.

**Fig. 2.**
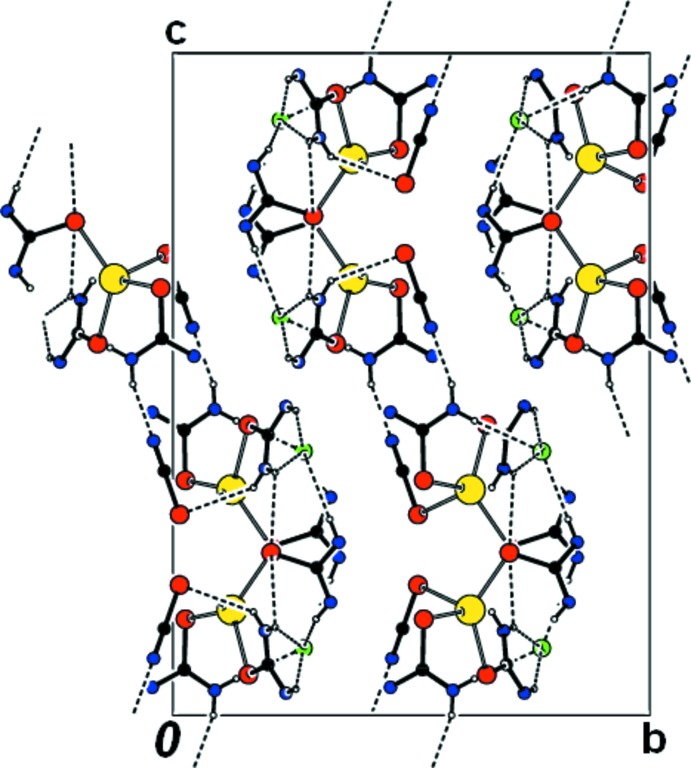
Packing diagram of (I) viewed along the a axis. Intramolecular N—H···S hydrogen bonds and weak N—H···Cl, and N—H···N intermolecular interactions are shown as dashed lines.

## supplementary crystallographic information

### Comment

This work is part of a research project concerning the investigation of thiourea (N_2_H_4_CS) and thiocyanate (SCN) based metal organic crystalline materials and their derivatives (Ramesh *et al.*, 2012). Transition metal thiourea and thiocyanate coordination complexes are candidate materials for device applications including their nonlinear optical properties. As ligands, both thiourea and thiocyanate are interesting due to their potential formation of metalcoordination complexes as they exhibit multifunctional coordination modes due to the presence of 'S' and 'N' donor atoms. With reference to the hard and soft acids and bases) concept (Ozutsmi *et al.*, 1989; Bell *et al.*, 2001), thesoft cations show a pronounced affinity for coordination with the softer ligands, while hard cations prefer coordination with harder ligands. Several crystallographic reports about mercury(II) complexes usually consist of discrete monomeric molecules with tetrahedral (somewhat distorted) coordination environments around mercury(II) (Nawaz *et al.*, 2010). Here, we report the synthesis and structure of the title salt, [(SC(2NH_2_))_3_(SCN^-^)Hg(^2+^]^+^ . Cl^-^,(I).

In (I), the Hg^2+^ ion is coordinated to three softer S atoms of thiourea and one softer S atom of a thiocyanate anion in addition to the isolated chlorine ion (Fig. 1). Intramolecular N—H···S hydrogen bonds are observed which form S(6) ring motifs (Bernstein *et al.*, 1995). Bond distances and angles are in agreement with those reported for related compounds (Safari *et al.*, 2009; Nawaz *et al.*, 2010). The S—Hg—S angles vary widely from 87.39 (5)° to 128.02 (4)°, indicative of a distorted tetrahedral arrangement. The SCN^-^ moiety is planar [to within 0.007 (1) Å] with the C—N and C—S bond lengths corresponding to the values intermediate between single and double bonds. The S2—C4—N7 unit is nearly linear with a bond angle of 177.9 (6)°. In the crystal, the ions are stabilized by weak N—H···Cl, and N—H···N intermolecular interactions (Table.1) which form a three-dimensional network (Fig. 2).

### Experimental

A mixture of thiourea, ammonium thiocyanate and mercury (II) choloride were dissolved in aqueous solution in the molar ratio 3:1:1 and thoroughly mixed for an hour to obtain a homogenous mixture. The solution was allowed to evaporate slowly at ambient temperature. Colourless single crystals suitable for single-crystal XRD were obtained in 12 days.

### Refinement

All H atoms were positioned geometrically with N—H = 0.86 Å and constrained to ride on their parent atoms with *U*_iso_(H)=1.2Ueq.

### Crystal data

**Table:**

[Hg(NCS)(CH_4_N_2_S)_3_]Cl	*F*(000) = 1968
*M**_r_* = 522.49	*D*_x_ = 2.281 Mg m^−^^3^
Orthorhombic, *P**b**c**a*	Mo *K*α radiation, λ = 0.71073 Å
Hall symbol: -P 2ac 2ab	Cell parameters from 5125 reflections
*a* = 8.2175 (3) Å	θ = 2.4–31.2°
*b* = 16.3257 (8) Å	µ = 10.83 mm^−^^1^
*c* = 22.6793 (10) Å	*T* = 293 K
*V* = 3042.6 (2) Å^3^	Block, colorless
*Z* = 8	0.30 × 0.25 × 0.20 mm

### Data collection

**Table:**

Bruker Kappa APEXII CCD diffractometer	5125 independent reflections
Radiation source: fine-focus sealed tube	3579 reflections with *I* > 2σ(*I*)
Graphite monochromator	*R*_int_ = 0.057
ω and φ scans	θ_max_ = 31.8°, θ_min_ = 2.5°
Absorption correction: multi-scan (*SADABS*; Sheldrick, 2004)	*h* = −12→6
*T*_min_ = 0.140, *T*_max_ = 0.221	*k* = −23→24
38987 measured reflections	*l* = −32→33

### Refinement

**Table:**

Refinement on *F*^2^	Primary atom site location: structure-invariant direct methods
Least-squares matrix: full	Secondary atom site location: difference Fourier map
*R*[*F*^2^ > 2σ(*F*^2^)] = 0.036	Hydrogen site location: inferred from neighbouring sites
*wR*(*F*^2^) = 0.082	H-atom parameters constrained
*S* = 1.05	*w* = 1/[σ^2^(*F*_o_^2^) + (0.0291*P*)^2^ + 7.0058*P*] where *P* = (*F*_o_^2^ + 2*F*_c_^2^)/3
5125 reflections	(Δ/σ)_max_ = 0.001
155 parameters	Δρ_max_ = 2.17 e Å^−^^3^
0 restraints	Δρ_min_ = −1.21 e Å^−^^3^

### Special details

**Table:**

Geometry. All e.s.d.'s (except the e.s.d. in the dihedral angle between two l.s. planes) are estimated using the full covariance matrix. The cell e.s.d.'s are taken into account individually in the estimation of e.s.d.'s in distances, angles and torsion angles; correlations between e.s.d.'s in cell parameters are only used when they are defined by crystal symmetry. An approximate (isotropic) treatment of cell e.s.d.'s is used for estimating e.s.d.'s involving l.s. planes.
Refinement. Refinement of *F*^2^ against ALL reflections. The weighted *R*-factor *wR* and goodness of fit *S* are based on *F*^2^, conventional *R*-factors *R* are based on *F*, with *F* set to zero for negative *F*^2^. The threshold expression of *F*^2^ > σ(*F*^2^) is used only for calculating *R*-factors(gt) *etc*. and is not relevant to the choice of reflections for refinement. *R*-factors based on *F*^2^ are statistically about twice as large as those based on *F*, and *R*- factors based on ALL data will be even larger.

### Fractional atomic coordinates and isotropic or equivalent isotropic displacement parameters (Å^2^)

**Table:**

	*x*	*y*	*z*	*U*_iso_*/*U*_eq_	
C1	1.2410 (5)	0.1993 (3)	0.42350 (17)	0.0280 (8)	
C2	1.0389 (5)	0.3016 (3)	0.2771 (2)	0.0321 (9)	
C3	0.7067 (6)	0.0234 (3)	0.43089 (19)	0.0333 (9)	
C4	1.2429 (7)	−0.0184 (3)	0.3691 (2)	0.0498 (13)	
N1	1.3113 (5)	0.1934 (3)	0.37245 (16)	0.0488 (12)	
H1A	1.4063	0.2142	0.3671	0.059*	
H1B	1.2628	0.1687	0.3439	0.059*	
N2	1.3169 (5)	0.2370 (3)	0.46633 (17)	0.0445 (10)	
H2A	1.4119	0.2575	0.4604	0.053*	
H2B	1.2718	0.2414	0.5004	0.053*	
N3	1.0072 (6)	0.3302 (3)	0.32922 (19)	0.0511 (11)	
H3A	1.0352	0.3794	0.3383	0.061*	
H3B	0.9582	0.2999	0.3547	0.061*	
N4	1.1131 (6)	0.3476 (3)	0.2386 (2)	0.0541 (12)	
H4A	1.1409	0.3967	0.2477	0.065*	
H4B	1.1342	0.3287	0.2040	0.065*	
N5	0.6681 (6)	0.0872 (3)	0.46201 (18)	0.0496 (11)	
H5A	0.6676	0.0844	0.4999	0.060*	
H5B	0.6430	0.1324	0.4448	0.060*	
N6	0.7440 (8)	−0.0438 (3)	0.4576 (2)	0.0667 (15)	
H6A	0.7430	−0.0458	0.4955	0.080*	
H6B	0.7698	−0.0865	0.4375	0.080*	
N7	1.2853 (8)	−0.0407 (4)	0.4142 (2)	0.0779 (18)	
Hg1	0.92567 (2)	0.123362 (12)	0.340864 (7)	0.03933 (7)	
S1	1.05319 (12)	0.15817 (7)	0.43857 (4)	0.0313 (2)	
S2	1.18864 (19)	0.01386 (9)	0.30317 (6)	0.0529 (4)	
S3	0.70350 (17)	0.02592 (8)	0.35487 (5)	0.0430 (3)	
S4	0.98486 (18)	0.20568 (7)	0.25448 (5)	0.0411 (3)	
Cl1	0.66976 (13)	0.27345 (7)	0.39858 (4)	0.0323 (2)	

### Atomic displacement parameters (Å^2^)

**Table:**

	*U*^11^	*U*^22^	*U*^33^	*U*^12^	*U*^13^	*U*^23^
C1	0.025 (2)	0.034 (2)	0.0245 (18)	0.0005 (16)	−0.0007 (15)	0.0005 (16)
C2	0.029 (2)	0.028 (2)	0.040 (2)	−0.0008 (16)	0.0019 (17)	−0.0016 (17)
C3	0.039 (3)	0.029 (2)	0.031 (2)	−0.0060 (18)	0.0015 (18)	0.0054 (17)
C4	0.060 (3)	0.044 (3)	0.046 (3)	0.022 (3)	0.006 (2)	−0.005 (2)
N1	0.032 (2)	0.090 (3)	0.0248 (18)	−0.021 (2)	0.0068 (15)	−0.010 (2)
N2	0.036 (2)	0.066 (3)	0.0310 (19)	−0.019 (2)	0.0034 (16)	−0.0137 (19)
N3	0.061 (3)	0.037 (2)	0.055 (3)	−0.013 (2)	0.019 (2)	−0.0159 (19)
N4	0.076 (3)	0.030 (2)	0.057 (3)	−0.015 (2)	0.024 (2)	−0.0046 (19)
N5	0.078 (3)	0.034 (2)	0.037 (2)	0.007 (2)	0.002 (2)	0.0028 (18)
N6	0.123 (5)	0.037 (3)	0.040 (2)	0.026 (3)	0.002 (3)	0.008 (2)
N7	0.110 (5)	0.079 (4)	0.044 (3)	0.043 (4)	0.005 (3)	0.008 (3)
Hg1	0.04559 (12)	0.04260 (11)	0.02981 (9)	−0.01684 (8)	−0.00241 (7)	0.00517 (7)
S1	0.0253 (5)	0.0458 (6)	0.0226 (4)	−0.0040 (4)	0.0020 (4)	−0.0053 (4)
S2	0.0672 (10)	0.0556 (8)	0.0358 (6)	0.0222 (7)	−0.0004 (6)	−0.0097 (6)
S3	0.0545 (8)	0.0436 (7)	0.0310 (5)	−0.0259 (6)	−0.0047 (5)	0.0018 (5)
S4	0.0683 (8)	0.0323 (6)	0.0227 (5)	−0.0161 (6)	−0.0053 (5)	0.0020 (4)
Cl1	0.0305 (5)	0.0362 (5)	0.0301 (5)	−0.0054 (4)	−0.0013 (4)	0.0004 (4)

### Geometric parameters (Å, º)

**Table:**

C1—N1	1.297 (5)	N2—H2B	0.8600
C1—N2	1.309 (5)	N3—H3A	0.8600
C1—S1	1.717 (4)	N3—H3B	0.8600
C2—N3	1.297 (6)	N4—H4A	0.8600
C2—N4	1.302 (6)	N4—H4B	0.8600
C2—S4	1.707 (4)	N5—H5A	0.8600
C3—N6	1.290 (6)	N5—H5B	0.8600
C3—N5	1.298 (6)	N6—H6A	0.8600
C3—S3	1.725 (4)	N6—H6B	0.8600
C4—N7	1.141 (7)	Hg1—S4	2.4250 (11)
C4—S2	1.647 (6)	Hg1—S3	2.4422 (12)
C4—S2	1.647 (6)	Hg1—S1	2.5162 (10)
N1—H1A	0.8600	Hg1—S2	2.9320 (14)
N1—H1B	0.8600	Hg1—S2	2.9320 (14)
N2—H2A	0.8600		
			
N1—C1—N2	119.0 (4)	C2—N4—H4B	120.0
N1—C1—S1	123.3 (3)	H4A—N4—H4B	120.0
N2—C1—S1	117.7 (3)	C3—N5—H5A	120.0
N3—C2—N4	119.9 (4)	C3—N5—H5B	120.0
N3—C2—S4	123.4 (4)	H5A—N5—H5B	120.0
N4—C2—S4	116.7 (4)	C3—N6—H6A	120.0
N6—C3—N5	119.1 (4)	C3—N6—H6B	120.0
N6—C3—S3	119.6 (4)	H6A—N6—H6B	120.0
N5—C3—S3	121.4 (4)	S4—Hg1—S3	128.02 (4)
N7—C4—S2	177.9 (6)	S4—Hg1—S1	120.18 (4)
N7—C4—S2	177.9 (6)	S3—Hg1—S1	110.11 (4)
C1—N1—H1A	120.0	S4—Hg1—S2	87.39 (5)
C1—N1—H1B	120.0	S3—Hg1—S2	101.05 (5)
H1A—N1—H1B	120.0	S1—Hg1—S2	95.02 (4)
C1—N2—H2A	120.0	S4—Hg1—S2	87.39 (5)
C1—N2—H2B	120.0	S3—Hg1—S2	101.05 (5)
H2A—N2—H2B	120.0	S1—Hg1—S2	95.02 (4)
C2—N3—H3A	120.0	C1—S1—Hg1	106.69 (14)
C2—N3—H3B	120.0	C4—S2—Hg1	97.45 (18)
H3A—N3—H3B	120.0	C3—S3—Hg1	97.71 (15)
C2—N4—H4A	120.0	C2—S4—Hg1	108.55 (16)
			
N1—C1—S1—Hg1	−14.6 (4)	S2—Hg1—S2—C4	0 (9)
N2—C1—S1—Hg1	166.6 (3)	N6—C3—S3—Hg1	−115.5 (4)
S4—Hg1—S1—C1	−32.03 (16)	N5—C3—S3—Hg1	66.4 (4)
S3—Hg1—S1—C1	161.59 (16)	S4—Hg1—S3—C3	−160.34 (16)
S2—Hg1—S1—C1	57.87 (16)	S1—Hg1—S3—C3	4.69 (17)
S2—Hg1—S1—C1	57.87 (16)	S2—Hg1—S3—C3	104.27 (17)
S2—C4—S2—Hg1	0 (100)	S2—Hg1—S3—C3	104.27 (17)
S4—Hg1—S2—S2	0.00 (8)	N3—C2—S4—Hg1	−17.9 (5)
S3—Hg1—S2—S2	0.00 (8)	N4—C2—S4—Hg1	163.4 (4)
S1—Hg1—S2—S2	0.00 (8)	S3—Hg1—S4—C2	138.25 (16)
S4—Hg1—S2—C4	150.5 (2)	S1—Hg1—S4—C2	−25.45 (17)
S3—Hg1—S2—C4	−81.2 (2)	S2—Hg1—S4—C2	−119.74 (17)
S1—Hg1—S2—C4	30.5 (2)	S2—Hg1—S4—C2	−119.74 (17)

### Hydrogen-bond geometry (Å, º)

**Table:**

*D*—H···*A*	*D*—H	H···*A*	*D*···*A*	*D*—H···*A*
N1—H1*A*···Cl1^i^	0.86	2.48	3.277 (4)	155
N1—H1*B*···S2	0.86	2.76	3.475 (5)	142
N2—H2*A*···Cl1^i^	0.86	2.55	3.335 (4)	152
N3—H3*B*···Cl1	0.86	2.61	3.320 (5)	141
N4—H4*B*···Cl1^ii^	0.86	2.51	3.370 (5)	175
N5—H5*B*···Cl1	0.86	2.54	3.363 (4)	161
N5—H5*A*···N7^iii^	0.86	2.11	2.933 (7)	160

Symmetry codes: (i) *x*+1, *y*, *z*; (ii) *x*+1/2, *y*, −*z*+1/2; (iii) −*x*+2, −*y*, −*z*+1.
